# Supplementary material for: Electrochemical Organophosphorus Pesticide Detection Using Nanostructured Gold-Modified Electrodes
Source: Sensors (Basel). 2022 Dec 16;22(24):9938. doi: 10.3390/s22249938 (PMC9787336; doi:10.3390/s22249938)
Supplement: Supplementary file 1 [file sensors-22-09938-s001.zip › sensors-2063281-supplementary.pdf]

sensors

MDPI

Article

Electrochemical Organophosphorus Pesticide Detection Using Nanostructured Gold-Modified Electrodes

Han-Wei Chang [1,2,*], Chien-Lin Chen [3], Yan-Hua Chen [1,2], Yu-Ming Chang [1,2], Feng-Jiin Liu [1,2] and Yu-Chen Tsai [3,*]

Supplementary Materials

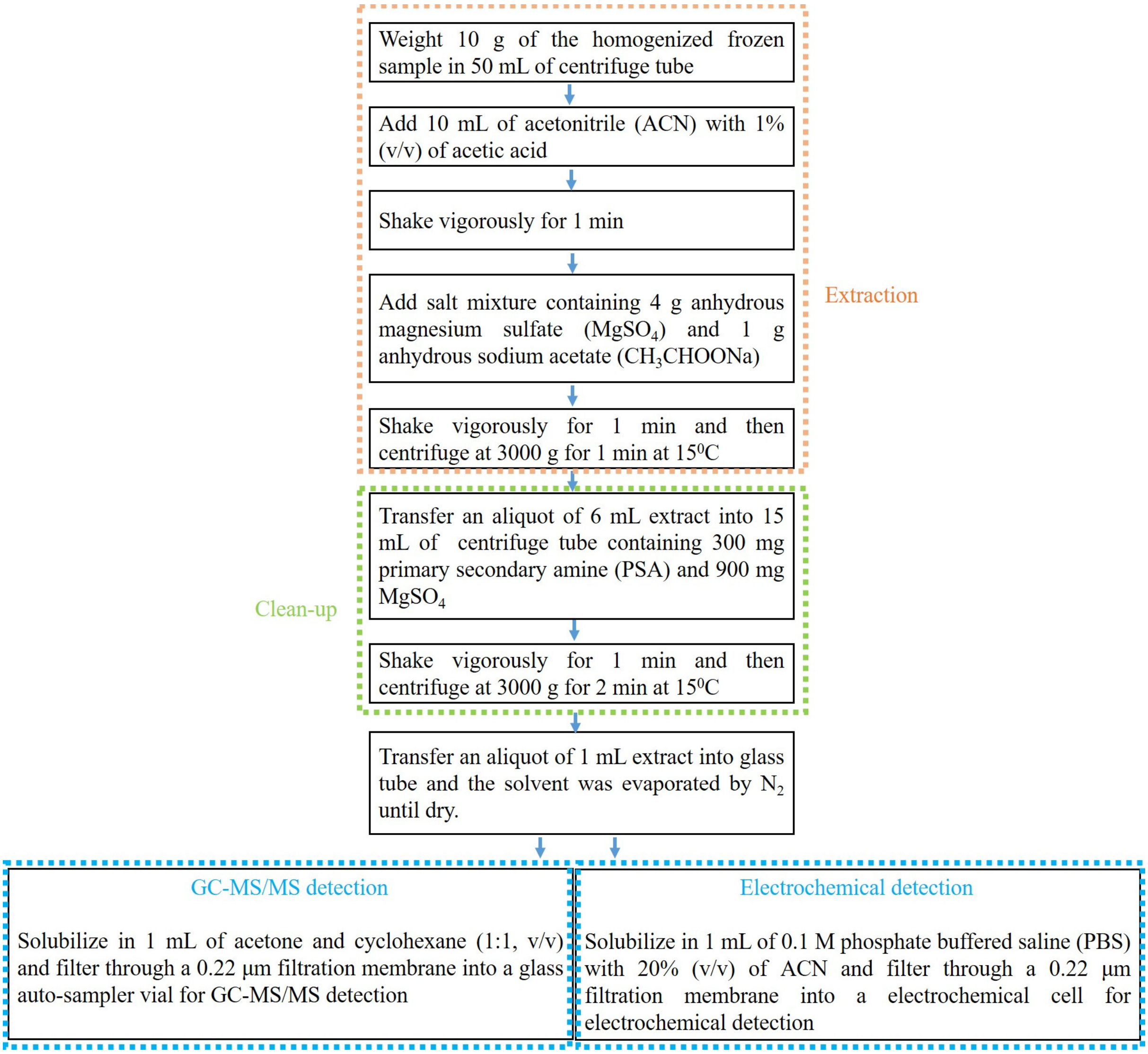

Figure S1. The extraction and cleanup steps of the QuEChERS method combined with a GC–MS/MS and electrochemistry.
